# Supplementary material for: Oxidative phosphorylation is a metabolic vulnerability of endocrine therapy and palbociclib resistant metastatic breast cancers
Source: Nat Commun. 2023 Jul 14;14:4221. doi: 10.1038/s41467-023-40022-5 (PMC10349040; doi:10.1038/s41467-023-40022-5)
Supplement: Supplementary file 1 — Supplementary Information [file 41467_2023_40022_MOESM1_ESM.pdf]

Supplemental information

Figure S1

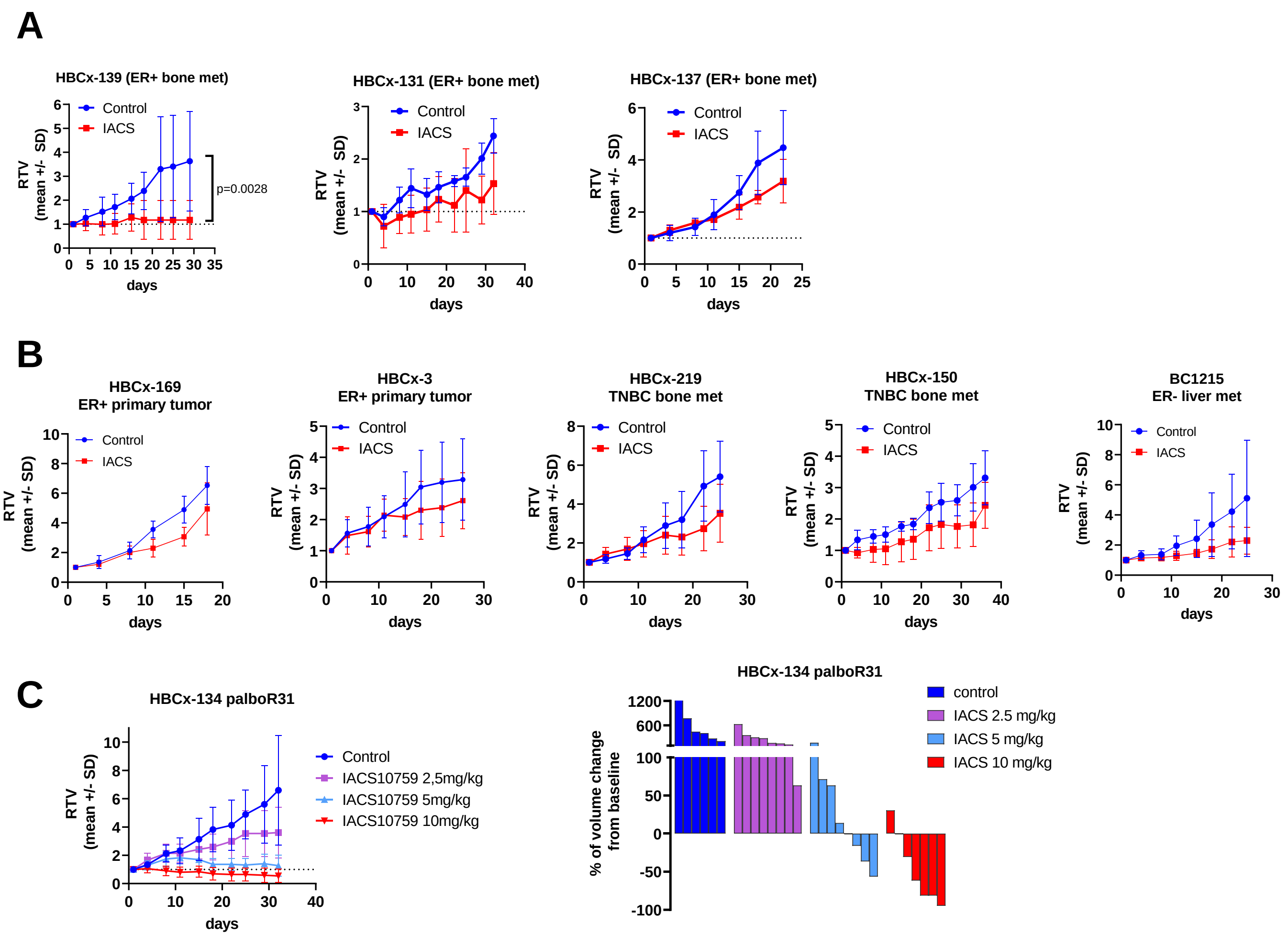

Two-way RM ANOVA					
Source of Variation	% of total variation	P value	P value summary		
Row Factor TIME x Column Factor IACS dose	19.13	<0,0001	****		
Row Factor TIME	12.42	<0,0001	****		
Column Factor IACS dose	35.25	<0,0001	****		
Subject	21.57	<0,0001	****		
Tukey's multiple comparisons test	Predicted (LS) mean diff,	95,00% CI of diff,	Below threshold?	Summary	Adjusted P Value
day 32					
Control vs. IACS10759 2,5mg/kg	2.985	1,480 to 4,490	Yes	****	<0,0001
Control vs. IACS10759 5mg/kg	5.328	3,823 to 6,833	Yes	****	<0,0001
Control vs. IACS10759 10mg/kg	6.05	4,500 to 7,600	Yes	****	<0,0001
IACS10759 2,5mg/kg vs. IACS10759 5mg/kg	2.344	0,9502 to 3,737	Yes	***	0.0001
IACS10759 2,5mg/kg vs. IACS10759 10mg/kg	3.065	1,623 to 4,508	Yes	****	<0,0001
IACS10759 5mg/kg vs. IACS10759 10mg/kg	0.7217	-0,7206 to 2,164	No	ns	0.5675

Figure S1: *in vivo* evaluation of IACS activity in PDX of bone ER positive metastasis **(A)**, liver metastasis, TNBC bone metastases and ER+ primary tumours **(B)** and in the Palbociclib-resistant HBCx-134 palboR31 PDX **(C)**. Mean +/- SD. HBCx-139: n= 7 (control and IACS); HBCx-131: n=3 (control) and n=5 (IACS); HBCx-137: n=5 (control and IACS); HBCx-169: n=5 (control and IACS); HBCx-3: n=8 (control) and n=9 (IACS); HBCx-219: n=6 (control and IACS); HBCx-150: n=4 (control) and n=5 (IACS); BC1215: n=4 (control and IACS). HBCx-131 palbo31: n=6 (control), n=8 (IACS 2.5 mg/kg and IACS 5 mg/kg), n=7 (IACS 10 mg/kg). The Turkey's multiple comparison test applied to the HBCx-134 palboR31 is shown under figure S1C (Two-way RM Anova). The difference between HBCx-139 control and treated group was analysed with the Mann Whitney test (p = 0.0028, two-tailed).

Figure S2

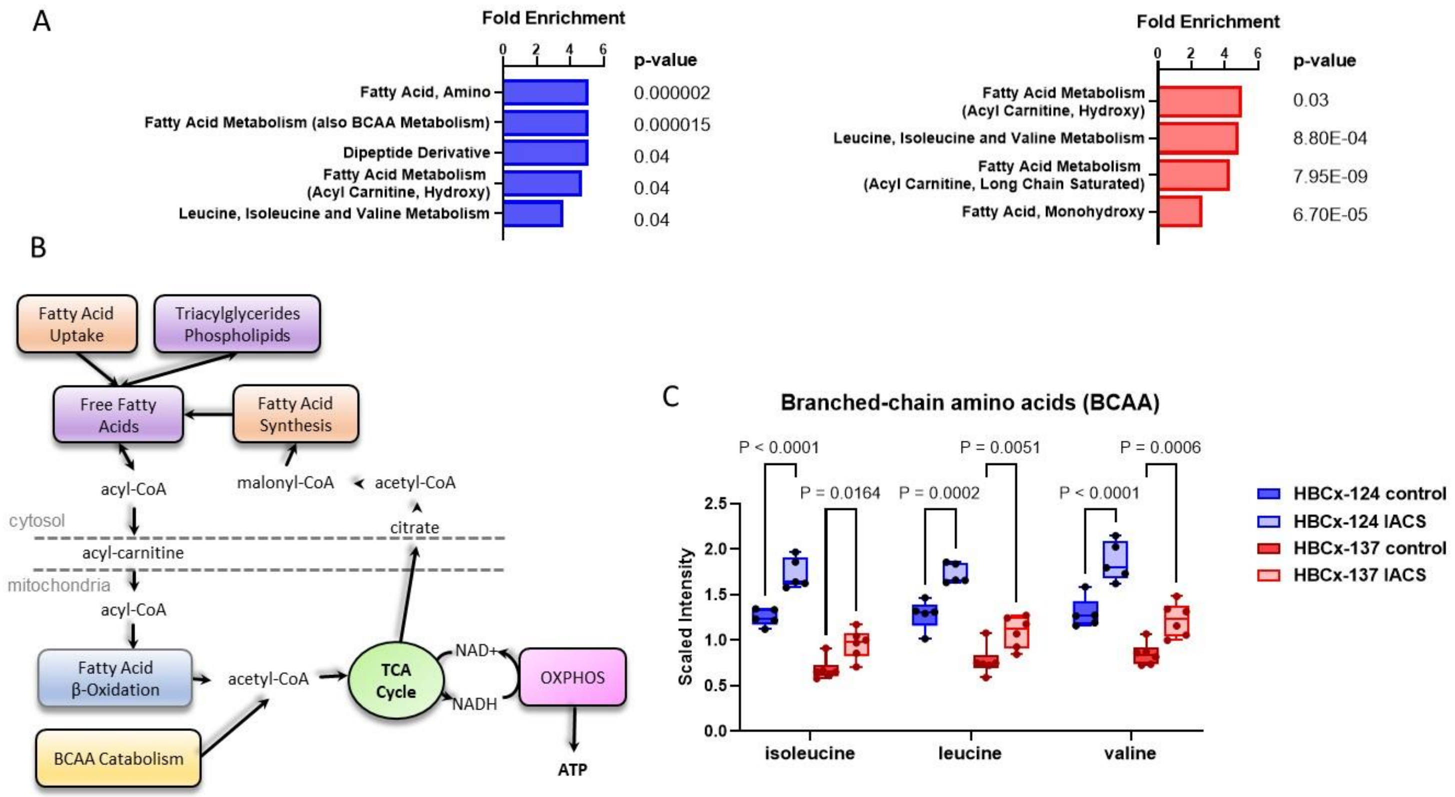

Figure S2. (A) Metabolic Pathway Enrichment Analysis of differentially expressed metabolites between IACS-treated and control xenografts of HBCx-124 and HBCx-137 PDX. p value were calculated with right-tailed Fisher's exact test (uncorrected). **(B)** Metabolic pathways showing the transport of fatty acids into mitochondria through the carnitine shuttle. In the mitochondria, these fatty acyl carnitines are progressively shortened by two carbons through rounds of β-oxidation, generating NADH, FADH2, and acetyl-CoA. **(C)** Min/Max Whiskers plots showing the levels of branched-chain amino acids (BCAA) in HBCx-124 and HBCx-137 PDX control and IACS treated groups. n= 5 (HBCx-124 control and treated groups) and n=6 (HBCx-137 control and treated groups)**.** Tukey's multiple comparisons test (2-way Anova).

Figure S3

A

	AIFM1	NDUFV1	NDUFAB1	NDUFA7	NDUFS6	MRPS12
AIFM1	1.00					
NDUFV1	0.52	1.00				
NDUFAB1	0.46	0.36	1.00			
NDUFA7	0.45	0.39	0.57	1.00		
NDUFS6	0.59	0.53	0.54	0.53	1.00	
MRSP12	0.65	0.63	0.52	0.53	0.66	1.00

B

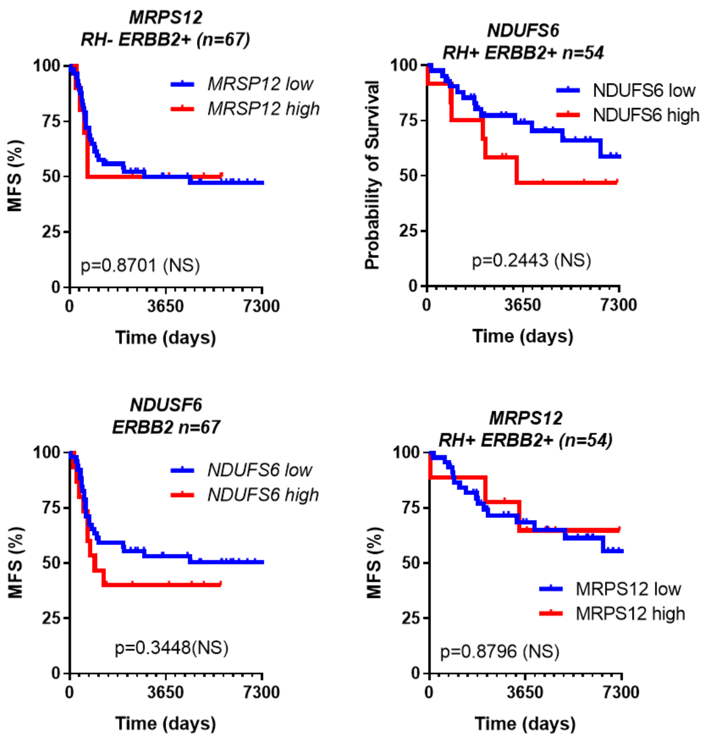

Figure S3. (A) Pearson correlation analysis of *AIFM1*, *NDUFV1*, *NDUFAB1*, *NDUFA7*, *NDUFS6* and *MRSP12* genes expression determined by RT-PCR analysis in 503 breast tumours. **(B)** Metastasis-free survival (MFS) of RH- ERBB2+ and RH+ ERBB2+ breast cancer patients according to *MRPS12* and *NDUFS6* low and high expression (optimal cut-off). P values were calculated with the Log-rank (Mantel-Cox) test.

Supplementary Tables

Table S1. Gene Set enrichment analysis of RNAseq data of IACS treated HBCx-124 xenografts as compared to control HBCx-124 xenografts. ES: enrichment score, NES: normalized enrichment score; NOM p val: nominal p value; FDR q value: p-value adjusted for the False Discovery Rate (FDR).

**GSEA REPORT FOR IACS TREATED HBCx-124 xenografts**					
**NAME**	SIZE	ES	NES	NOM p-val	FDR q-val
REACTOME_ACTIVATION_OF_THE_MRNA_UPON_BINDING_OF_THE_CAP_BINDING_COMPLEX_AND_EIFS_AND_SUBSEQUENT_BINDING_TO_43S	26	0.632	2.092	0.000	0.052
REACTOME_RESPONSE_OF_EIF2AK4_GCN2_TO_AMINO_ACID_DEFICIENCY	15	0.658	1.944	0.003	0.154
REACTOME_EUKARYOTIC_TRANSLATION_INITIATION	34	0.552	1.911	0.000	0.155
REACTOME_FOXO_MEDIATED_TRANSCRIPTION_OF_CELL_DEATH_GENES	15	0.668	1.848	0.000	0.234
KEGG_ETHER_LIPID_METABOLISM	18	0.626	1.843	0.000	0.197
REACTOME_RESPONSE_OF_EIF2AK1_HRI_TO_HEME_DEFICIENCY	15	0.650	1.837	0.005	0.177
MED16_TARGET_GENES	24	0.567	1.817	0.000	0.187
REACTOME_PEROXISOMAL_PROTEIN_IMPORT	57	0.454	1.814	0.000	0.170
REACTOME_ATTENUATION_PHASE	25	0.535	1.772	0.005	0.228
KEGG_OTHER_GLYCAN_DEGRADATION	15	0.607	1.765	0.010	0.218
REACTOME_AMINO_ACIDS_REGULATE_MTORC1	50	0.461	1.761	0.000	0.205
REACTOME_FOXO_MEDIATED_TRANSCRIPTION	51	0.453	1.751	0.004	0.208
**GSEA REPORT FOR CONTROL HBCx-124 xenografts**					
**NAME**	SIZE	ES	NES	NOM p-val	FDR q-val
HSD17B8_TARGET_GENES	486	-0.734	-3.173	0.000	0.000
HALLMARK_E2F_TARGETS	195	-0.763	-3.096	0.000	0.000
HALLMARK_G2M_CHECKPOINT	186	-0.753	-2.995	0.000	0.000
REACTOME_CELL_CYCLE_CHECKPOINTS	241	-0.692	-2.839	0.000	0.000
REACTOME_MITOTIC_SPINDLE_CHECKPOINT	104	-0.764	-2.817	0.000	0.000
REACTOME_RESOLUTION_OF_SISTER_CHROMATID_COHESION	114	-0.745	-2.814	0.000	0.000
REACTOME_RHO_GTPASES_ACTIVATE_FORMINS	128	-0.707	-2.724	0.000	0.000
REACTOME_CELL_CYCLE_MITOTIC	472	-0.634	-2.697	0.000	0.000
PID_AURORA_B_PATHWAY	36	-0.838	-2.649	0.000	0.000
REACTOME_MITOTIC_PROMETAPHASE	189	-0.661	-2.645	0.000	0.000
REACTOME_MITOTIC_METAPHASE_AND_ANAPHASE	219	-0.647	-2.637	0.000	0.000
REACTOME_SEPARATION_OF_SISTER_CHROMATIDS	175	-0.664	-2.634	0.000	0.000
PID_PLK1_PATHWAY	41	-0.833	-2.620	0.000	0.000
PID_E2F_PATHWAY	70	-0.729	-2.607	0.000	0.000
PID_ATR_PATHWAY	38	-0.795	-2.574	0.000	0.000
RB_P107_DN.V1_UP	115	-0.678	-2.573	0.000	0.000
REACTOME_ACTIVATION_OF_ATR_IN_RESPONSE_TO_REPLICATION_STRESS	37	-0.804	-2.558	0.000	0.000
REACTOME_MITOTIC_G1_PHASE_AND_G1_S_TRANSITION	141	-0.648	-2.525	0.000	0.000
KEGG_CELL_CYCLE	117	-0.668	-2.524	0.000	0.000
REACTOME_DNA_REPLICATION	123	-0.660	-2.511	0.000	0.000
REACTOME_DEPOSITION_OF_NEW_CENPA_CONTAINING_NUCLEOSOMES_AT_THE_CENTROMERE	24	-0.858	-2.503	0.000	0.000
REACTOME_G2_M_CHECKPOINTS	126	-0.648	-2.471	0.000	0.000
REACTOME_G1_S_SPECIFIC_TRANSCRIPTION	28	-0.838	-2.453	0.000	0.000
REACTOME_M_PHASE	334	-0.583	-2.452	0.000	0.000

Table S2. Gene Set enrichment analysis of RNAseq data of IACS resistant PDX as compared to IACS responder PDX. ES: enrichment score, NES: normalized enrichment score; NOM p val: nominal p value; FDR q value: p-value adjusted for the False Discovery Rate (FDR).

**GSEA REPORT FOR IACS RESPONDERS**					
NAME	SIZE	ES	NES	NOM p-val	FDR q-val
HALLMARK_INTERFERON_ALPHA_RESPONSE	94	-0.488	-2.271	0.000	0.000
HALLMARK_ESTROGEN_RESPONSE_EARLY	194	-0.400	-2.089	0.000	0.000
HALLMARK_INTERFERON_GAMMA_RESPONSE	184	-0.356	-1.864	0.000	0.004
HALLMARK_ESTROGEN_RESPONSE_LATE	195	-0.342	-1.802	0.000	0.005
HALLMARK_PI3K_AKT_MTOR_SIGNALING	97	-0.366	-1.726	0.000	0.008
HALLMARK_XENOBIOTIC_METABOLISM	179	-0.320	-1.691	0.000	0.009
HALLMARK_PROTEIN_SECRETION	93	-0.351	-1.628	0.000	0.014
HALLMARK_OXIDATIVE_PHOSPHORYLATION	183	-0.313	-1.626	0.004	0.013
HALLMARK_ADIPOGENESIS	183	-0.301	-1.573	0.000	0.018
HALLMARK_IL6_JAK_STAT3_SIGNALING	74	-0.302	-1.376	0.048	0.095
HALLMARK_FATTY_ACID_METABOLISM	148	-0.257	-1.309	0.041	0.149

GSEA REPORT FOR IACS RESISTANT					
NAME	SIZE	ES	NES	NOM p-val	FDR q-val
HALLMARK_E2F_TARGETS	195	0.358	1.830	0.000	0.007
HALLMARK_G2M_CHECKPOINT	188	0.349	1.791	0.000	0.005
HALLMARK_ANGIOGENESIS	30	0.458	1.667	0.015	0.013
HALLMARK_PANCREAS_BETA_CELLS	30	0.468	1.664	0.006	0.010
HALLMARK_EPITHELIAL_MESENCHYMAL_TRANSITION	188	0.307	1.593	0.000	0.018
HALLMARK_MITOTIC_SPINDLE	197	0.298	1.541	0.002	0.023
HALLMARK_SPERMATOGENESIS	107	0.314	1.465	0.007	0.045

Table S3. Characteristics of the 503 breast cancers

	Number of patients	% patients	Number with metastases	% metastases	p value[e]
Total	503	100.0%	202	40.16%	
Age					
≤50	117	23.26%	51	25.25%	
>50	386	76.74%	151	74.75%	0.2895 (NS)
SBR histological grade[a,b]					
I	58	11.84%	12	5.94%	
II	231	47.14%	97	48.02%	**0.0036**
III	201	41.02%	93	46.04%	
Lymph node status [c]					
0	149	29.86%	46	22.77%	
1-3	242	48.50%	85	42.08%	**<0.0001**
>3	108	21.64%	71	35.15%	
Macroscopic tumor size[d]					
<25mm	237	48.07%	74	36.82%	
>25mm	256	51.93%	127	63.18%	**<0.0001**
ERα status					
Negative	169	33.6%	72	35.64%	
Positive	334	66.4%	130	64.36%	0.0845 (NS)
PR status					
Negative	241	47.91%	104	51.49%	
Positive	262	52.09%	98	48.51%	**0.0349**
ERBB2 status					
Negative	382	75.94%	149	73.76%	
Positive	121	24.06%	53	26.24%	0.2246 (NS)
Molecular subtypes					
HR- ERBB2-	95	18.89%	36	17.82%	
HR- ERBB2+	67	13.32%	34	16.83%	
HR+ ERBB2-	287	57.06%	113	55.94%	**0.0484**
HR+ ERBB2+	54	10.73%	19	9.41%	

Table S3 Characteristics of the 503 breast cancers. **a** Scarff Bloom Richardson classification, **b** Information available for 490 patients, **c** Information available for 499 patients, **d** Information available for 493 patients, **e** Metastasis-free survival (Log-rank Mantel-Cox test)

Table S4

	Total population (%)	***Low MRPS12***	***High MRPS12***	*p-value*	*Total population (%)*	***Low NDUFS6***	***High NDUFS6***	*p-value [a]*
	Number of patients (%)				Number of patients (%)			
Total	503 (100)	443 (88.07)	60 (11.93)		503 (100)	419 (83.3)	84 (16.7)	
Age								
≤50	117 (23.26)	99 (84.62)	18 (15.38)	0.1880	117 (23.26)	94 (80.34)	23 (19.66)	0.3274
>50	386 (76.74)	344 (89.12)	42 (10.88)		386 (76.74)	325 (84.2)	61 (15.80)	
SBR histological grade *b.c*								
I	58 (11.84)	57 (98.28)	1 (1.72)	**0.0002**	58 (11.84)	56 (96.55)	2 (3.45)	**0.0004**
II	231 (47.14)	211 (91.34)	20 (8.66)		231 (47.14)	198 (85.71)	33 (14.29)	
III	201 (41.02)	164 (81.59)	37 (18.41)		201 (41.02)	153 (76.12)	48 (23.88)	
Lymph node status *d*								
0	149 (29.86)	129 (86.58)	20 (13.42)	0.7995	149 (29.86)	126 (84.56)	23 (15.44)	0.8627 (NS)
1-3	242 (48.5)	215 (88.84)	27 (11.16)		242 (48.50)	200 (82.64)	42 (17.36)	
>3	108 (21.64)	95 (87.96)	13 (12.04)		108 (21.64)	91 (84.26)	17 (15.74)	
Macroscopic tumor size *e*								
≤ 25mm	237 (48.07)	217 (91.56)	20 (8.44)	**0.0202**	237 (48.07)	203 (85.65)	34 (14.35)	0.1895
> 25mm	256 (51.93)	217 (84.77)	39 (15.23)		256 (51.93)	208 (81.25)	48 (18.75)	
ERα status								
Negative	169 (33.6)	136 (80.47)	33 (19.53)	**0.0002**	169 (33.6)	128 (75.74)	41 (24.26)	**0.0012**
Positive	334 (66.4)	307 (91.92)	27 (8.08)		334 (66.4)	291 (87.13)	43 (12.87)	
PR status								
Negative	241 (47.91)	203 (84.23)	38 (15.77)	**0.0108**	241 (47.91)	189 (78.42)	52 (21.58)	**0.0049**
Positive	262 (52.09)	240 (91.60)	22 (8.40)		262 (52.09)	230 (87.79)	32 (12.21)	
ERBB2 status								
Negative	382 (75.94)	341 (89.27)	41 (10.73)	0.1416	382 (75.94)	325 (85.08)	57 (14.92)	0.0574
Positive	121 (24.06)	102 (84.30)	19 (15.70)		121 (24.06)	94 (77.69)	27 (22.31)	
Molecular subtypes								
HR- ERBB2-	95 (18.89)	73 (76.84)	22 (23.16)	**0.0001**	95 (18.89)	71 (74.74)	24 (25.26)	**0.0040**
HR- ERBB2+	67 (13.32)	57 (85.07)	10 (14.93)		67 (13.32)	52 (77.61)	15 (22.39)	
HR+ ERBB2-	287 (57.06)	268 (93.38)	19 (6.62)		287 (57.06)	254 (88.50)	33 (11.50)	
HR+ ERBB2+	54 (10.73)	45 (83.33)	9 (16.67)		54 (10.73)	42 (77.78)	12 (22.22)	
MKI67 mRNA expression *h.j*								
median	8.35 (0.28-79.79)	7.54 (0.28-79.79)	21.64 (2.25-57.67)	**<0.0001**	8.35 (0.28-79.79)	7.48 (0.28-79.79)	16.13 (2.21-71.97)	**<0.0001**
Metastasis								
No	301 (59.84)	274 (91.03)	27 (8.97)	**0.0125**	301 (59.84)	260 (86.38)	41 (13.62)	**0.0238**
Yes	202 (40.16)	169 (83.66)	33 (16.34)		202 (40.16)	159 (78.71)	43 (21.29)	

Table S4: Relationship between NDUFS6 and MRPS12 transcripts levels and classical clinical biological parameters in a series of 503 breast cancer. NS: not significant; [a] Chi-squared Test (one-sided); [b] Scarff Bloom Richardson classification; [c] Information available for 490 patients; [d] Information available for 499 patients; [e] Information available for 493 patients; [f] Information available for 500 patients; [h] Information available for 502 patients; i Information available for 428 patients; [j] Mann Whitney Test (two-tailed).

Table S5: sequences of primers used in the RT-PCR analysis of OXPHOS associated genes

Gene	Upper primer (5' to 3')	Lower primer (5' to 3')
PDCD8 (AIFM1)	CTCTAACATCTGGGTGGCAGGA	ACTCACAACAGCGTGATCATGG
NDUFV1	CCCGCAGACGTGGGAGTGTT	TACCTCCACGGCGGCAGATT
NDUFAB1	GTTTGGACCAAGTGGAGATTATCAT	TGTGGACACATTAACTTTTCAGCAT
NDUFA7	GCTGCAGCTACGCTACCAGGAGAT	ATTGTTGGAGAGCTTGTGGCTAGGA
NDUFS6	CACACTGGCCAGGTTTATGATGAT	TGGCAAAGTTTTCATTCACCTCTT
MRPS12	CCCTAACTTGTGGCCCAGCTCT	CAGGCGGTGCATCTGGTTCA
TBP	TGCACAGGAGCCAAGAGTGAA	CACATCACAGCTCCCCACCA
