# Supplementary material for: Oxidative phosphorylation is a metabolic vulnerability of endocrine therapy and palbociclib resistant metastatic breast cancers
Source: Nat Commun. 2023 Jul 14;14:4221. doi: 10.1038/s41467-023-40022-5 (PMC10349040; doi:10.1038/s41467-023-40022-5)
Supplement: Supplementary file 4 — Description of Additional Supplementary Files [file 41467_2023_40022_MOESM4_ESM.pdf]

Description of Additional Supplementary Files

Supplementary Data 1
Description: Heat map of statistically significant biochemicals between PDX and patients' primary tumours (PT).

Supplementary Data 2
Description: Heat map of statistically significant biochemicals between HBCx-124 and HBCx-137 control and treated groups.

Supplementary Data 3
Description: Chemical annotation, sample meta data and normalized metabolomics data.
